# Spatial Chromatin Accessibility Analysis of Intratumor Heterogeneity in Breast Cancer

**DOI:** 10.1093/gpbjnl/qzag001

**Published:** 2026-01-12

**Authors:** Yingying Qian (钱莹莹), Miao Zhu (朱淼), Chongyang Ren (任重阳), Yeyong Zhou (周业永), Jian Xu (徐剑), Liang Dong (董良), Guangyu Zhang (张广雨), Cheukfai Li (李焯辉), Jiaoyi lv (吕娇怡), Qiaorui Xing (邢巧瑞), Guochun Zhang (张国淳), Guangdun Peng (彭广敦), Ning Liao (廖宁)

**Affiliations:** Division of Life Sciences and Medicine, University of Science and Technology of China, Hefei 230026, China; Center for Cell Lineage Technology and Engineering, Guangdong Provincial Key Laboratory of Stem Cell and Regenerative Medicine, Guangdong-Hong Kong Joint Laboratory for Stem Cell and Regenerative Medicine, GIBH-CUHK Joint Research Laboratory on Stem Cell and Regenerative Medicine, GIBH-HKU Guangdong-Hong Kong Stem Cell and Regenerative Medicine Research Centre, Guangzhou Institutes of Biomedicine and Health, Chinese Academy of Sciences, Guangzhou 510530, China; Center for Cell Lineage Technology and Engineering, Guangdong Provincial Key Laboratory of Stem Cell and Regenerative Medicine, Guangdong-Hong Kong Joint Laboratory for Stem Cell and Regenerative Medicine, GIBH-CUHK Joint Research Laboratory on Stem Cell and Regenerative Medicine, GIBH-HKU Guangdong-Hong Kong Stem Cell and Regenerative Medicine Research Centre, Guangzhou Institutes of Biomedicine and Health, Chinese Academy of Sciences, Guangzhou 510530, China; University of Chinese Academy of Sciences, Beijing 100049, China; Department of Breast Surgery, Guangdong Provincial People’s Hospital and Guangdong Academy of Medical Sciences, Guangzhou 510080, China; Center for Cell Lineage Technology and Engineering, Guangdong Provincial Key Laboratory of Stem Cell and Regenerative Medicine, Guangdong-Hong Kong Joint Laboratory for Stem Cell and Regenerative Medicine, GIBH-CUHK Joint Research Laboratory on Stem Cell and Regenerative Medicine, GIBH-HKU Guangdong-Hong Kong Stem Cell and Regenerative Medicine Research Centre, Guangzhou Institutes of Biomedicine and Health, Chinese Academy of Sciences, Guangzhou 510530, China; University of Chinese Academy of Sciences, Beijing 100049, China; School of Basic Medical Sciences, Guangzhou Medical University, Guangzhou 511436, China; Guangzhou National Laboratory, Guangzhou 510005, China; Center for Cell Lineage Technology and Engineering, Guangdong Provincial Key Laboratory of Stem Cell and Regenerative Medicine, Guangdong-Hong Kong Joint Laboratory for Stem Cell and Regenerative Medicine, GIBH-CUHK Joint Research Laboratory on Stem Cell and Regenerative Medicine, GIBH-HKU Guangdong-Hong Kong Stem Cell and Regenerative Medicine Research Centre, Guangzhou Institutes of Biomedicine and Health, Chinese Academy of Sciences, Guangzhou 510530, China; Center for Cell Lineage Technology and Engineering, Guangdong Provincial Key Laboratory of Stem Cell and Regenerative Medicine, Guangdong-Hong Kong Joint Laboratory for Stem Cell and Regenerative Medicine, GIBH-CUHK Joint Research Laboratory on Stem Cell and Regenerative Medicine, GIBH-HKU Guangdong-Hong Kong Stem Cell and Regenerative Medicine Research Centre, Guangzhou Institutes of Biomedicine and Health, Chinese Academy of Sciences, Guangzhou 510530, China; Department of Breast Surgery, Guangdong Provincial People’s Hospital and Guangdong Academy of Medical Sciences, Guangzhou 510080, China; Department of Breast Surgery, Guangdong Provincial People’s Hospital and Guangdong Academy of Medical Sciences, Guangzhou 510080, China; Center for Cell Lineage Technology and Engineering, Guangdong Provincial Key Laboratory of Stem Cell and Regenerative Medicine, Guangdong-Hong Kong Joint Laboratory for Stem Cell and Regenerative Medicine, GIBH-CUHK Joint Research Laboratory on Stem Cell and Regenerative Medicine, GIBH-HKU Guangdong-Hong Kong Stem Cell and Regenerative Medicine Research Centre, Guangzhou Institutes of Biomedicine and Health, Chinese Academy of Sciences, Guangzhou 510530, China; Department of Breast Surgery, Guangdong Provincial People’s Hospital and Guangdong Academy of Medical Sciences, Guangzhou 510080, China; Center for Cell Lineage Technology and Engineering, Guangdong Provincial Key Laboratory of Stem Cell and Regenerative Medicine, Guangdong-Hong Kong Joint Laboratory for Stem Cell and Regenerative Medicine, GIBH-CUHK Joint Research Laboratory on Stem Cell and Regenerative Medicine, GIBH-HKU Guangdong-Hong Kong Stem Cell and Regenerative Medicine Research Centre, Guangzhou Institutes of Biomedicine and Health, Chinese Academy of Sciences, Guangzhou 510530, China; University of Chinese Academy of Sciences, Beijing 100049, China; Department of Breast Surgery, Guangdong Provincial People’s Hospital and Guangdong Academy of Medical Sciences, Guangzhou 510080, China

**Keywords:** Breast cancer, Spatial ATAC-seq, Intratumoral heterogeneity, Tumor subclone, Tumor microenvironment

## Abstract

Intratumoral heterogeneity (ITH) is a major driver of mortality in breast cancer (BC) patients and a critical factor in the variable therapeutic outcomes observed in BC treatment. Understanding the mechanisms underlying ITH is essential for advancing both clinical and basic BC research. Chromatin accessibility is critical for the regulation of gene expression and cellular identity and plays a central role in shaping ITH and tumor evolution. However, studying chromatin accessibility *in situ* has been challenging due to the limited availability of technical platforms. Here, we leveraged the spatial assay for transposase-accessible chromatin sequencing (ATAC-seq) platform to profile the chromatin accessibility landscape of tumors from six BC patients. Our analyses revealed prominent heterogeneity within tumor regulatory modules and spatial variations in immune cell composition and stromal structures, offering a framework for the investigation of the molecular architecture underlying ITH. Moreover, we identified two tumor subclones with a potential common origin but distinct immune infiltration conferred by regulatory cascades, suggesting that epigenetic regulation may further contribute to the divergent tumor microenvironments and phenotypic diversity of these subclones. Our study provides novel insights into the molecular mechanisms driving ITH and opens up potential avenues for therapeutic intervention.

## Graphical abstract

**Figure:**
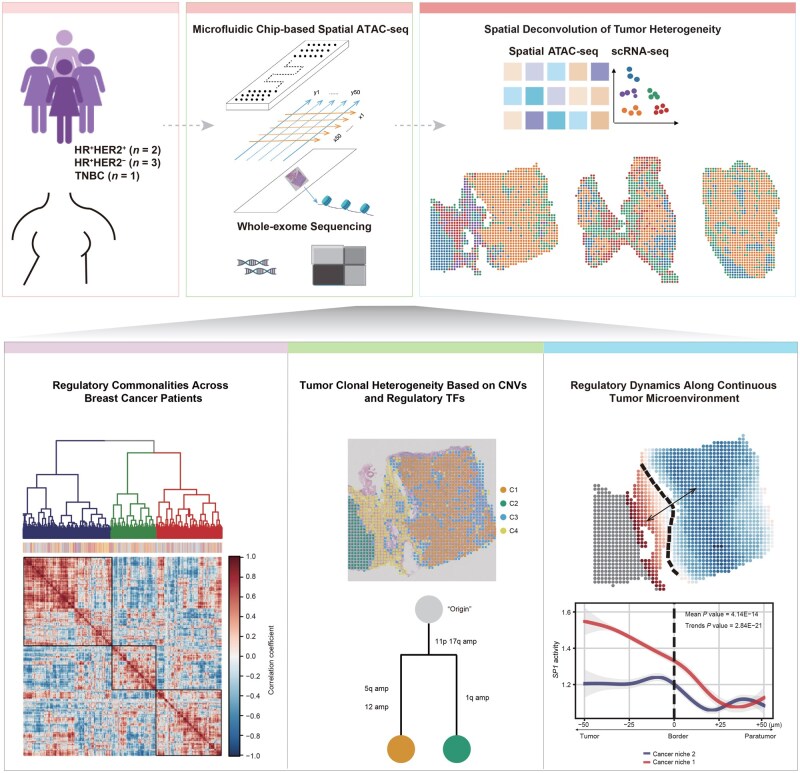

## Introduction

Breast cancer (BC) remains one of the most prominent health challenges faced by women globally [1]. Despite improvements in screening tests, diagnosis, and treatment, the mortality rate of BC remains a substantial public health concern. Traditionally, BC has not been considered an immunogenic disease, but an increased presence of tumor-infiltrating lymphocytes is consistently associated with a favorable BC prognosis [2]. Elevated tumor-associated lymphocyte levels have been observed in triple-negative breast cancer (TNBC) and human epidermal growth factor receptor 2 (HER2) positive breast cancer, suggesting that they are more immunogenic than other BC subtypes [3]. Consequently, it is urgent to discover and comprehensively explore the fundamental mechanisms that define BC molecular architecture and tumor microenvironment (TME).

BC is highly heterogeneous, making it difficult to attain optimal and consistent treatment with commonly available drugs or interventions. This persistent challenge is largely attributed to the intricate nature of the disease, which is characterized by both intertumoral heterogeneity and intratumoral heterogeneity (ITH) [4]. Intertumoral heterogeneity has been observed in distinct patient subgroups with markedly different genetic backgrounds, histopathological features, and clinical behaviors [5,6]. For example, luminal A patients respond well to hormonal therapies, while TNBC patients require more aggressive treatment due to their distinct biological features. However, ITH has only been recognized and gained traction in the field of oncology within the past few decades [7]. ITH originates from the genetic heterogeneity of cancer cells within a single tumor, leading to the coexistence of various cell populations exhibiting distinct phenotypic behaviors. Such diversity within tumors contributes to a complex TME, which significantly influences tumor progression, treatment response, and the potential for metastasis [8–10]. Therefore, a comprehensive understanding of the heterogeneous landscapes of tumors and their TME is pivotal for developing strategies aimed at combating resistance and enhancing treatment efficacy.

The development of sequencing technologies, particularly single-cell [11–13] and spatial transcriptomics sequencing [14–16], has transformed our capacity to analyze the dynamic interplay of cells and has unveiled new insights regarding the molecular landscape of the breast tumor ecosystem. In addition to gene expression, chromatin accessibility is crucial for regulating gene expression and cellular identity. Variations in accessibility revealed by single-cell assay for transposase-accessible chromatin using sequencing (ATAC-seq) analysis have been associated with the origin of intratumoral heterogeneity [16–19]. However, cells in multicellular tumor tissues progressively acquire specialized gene functions by coordinating *in situ* interaction between regulatory elements and transcription, which remains largely undocumented. To address this gap, the development of spatial ATAC-seq technology has emerged as a pivotal advancement. Spatial ATAC-seq data analysis has enabled the estimation of chromatin accessibility, *cis*-regulatory element activity, and transcription factor (TF) motif enrichment in a spatially dependent context [20], providing an unprecedented opportunity to dissect the complex cellular mechanisms underlying tumor progression, resistance, and metastasis.

Despite its potential, the application of spatial ATAC-seq methodologies in BC research has not yet been fully explored, primarily due to the limited availability of technical platforms [21]. Previously, we demonstrated the feasibility of obtaining high-quality spatial chromatin accessibility mapping in the developing mouse brain [22]. In this study, we investigated the tissue-wide chromatin accessibility in six BC patients by employing spatial ATAC-seq. Our findings highlighted prominent heterogeneity across tumor regulatory modules, immune cell infiltration, and stromal compositions, providing valuable biological insights into BC dynamics. We further revealed the spatial distribution and TME-dependent cellular interactions of tumor clones with distinct copy number variations (CNVs) across tumor regions, offering valuable insights into clonal evolution and expansion. The exploration of spatial ATAC-seq in the context of BC offers a valuable framework for comprehensively examining the fundamental mechanisms that regulate the molecular profiles of cancer cells and their interactions with the surrounding microenvironment.

## Results

### Overview of the experimental and analytical workflow

**Figure 1:**
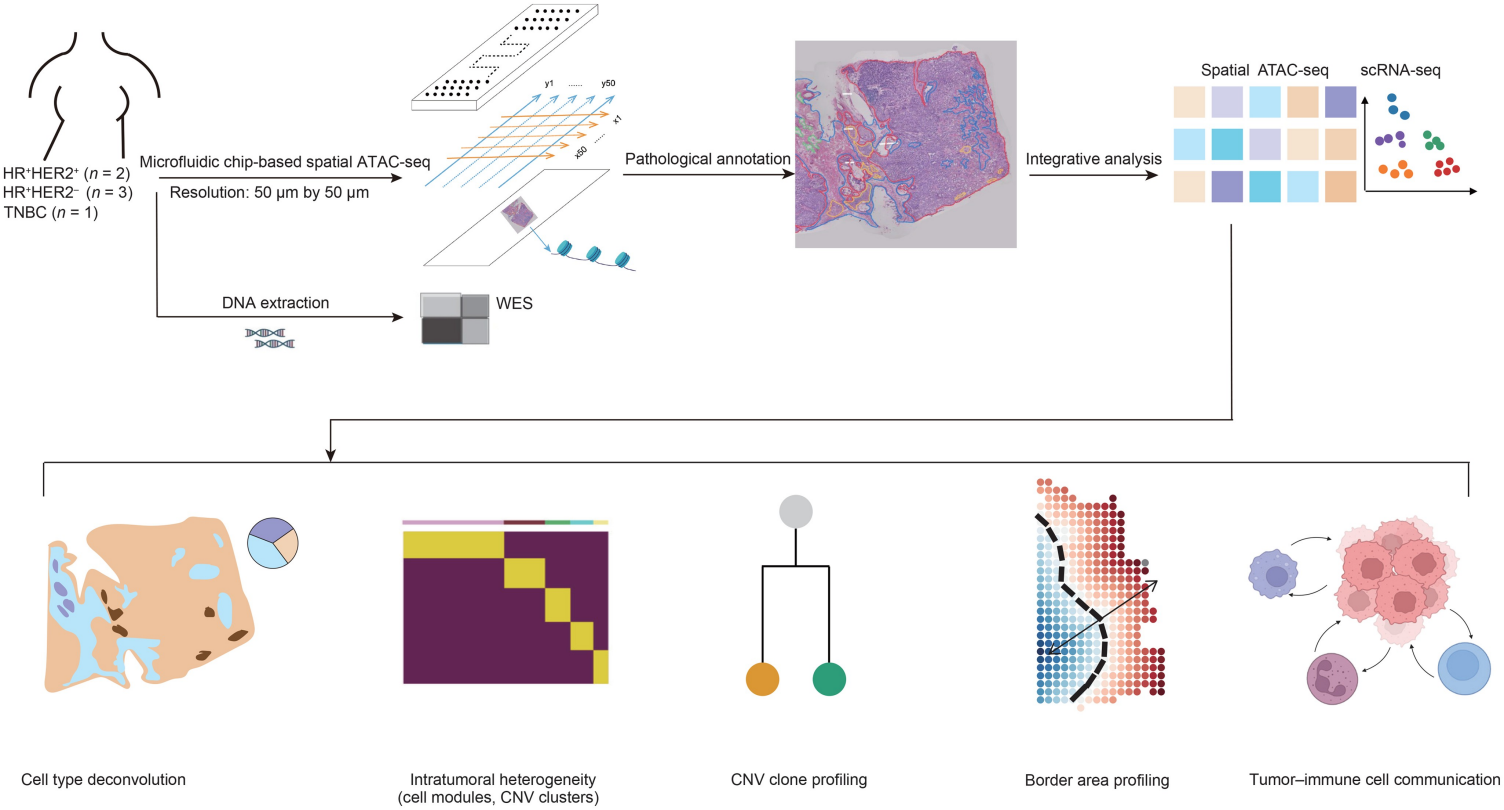
A summary of the study The workflow of this study includes sample collection, spatial ATAC-seq, WES, and data analysis. ATAC-seq, assay for transposase-accessible chromatin using sequencing; HR, hormone receptor; HER2, human epidermal growth factor receptor 2; TNBC, triple-negative breast cancer; scRNA-seq, single-cell RNA sequencing; CNV, copy number variation; WES, whole-exome sequencing.

To analyze the spatial heterogeneity of chromatin accessibility of three BC subtypes *in situ*, we collected tissue specimens from six patients (BC1–BC6) diagnosed with invasive ductal carcinoma, including two HR^+^HER2^+^ (BC1 and BC3), three HR^+^HER2^−^ (BC2, BC4, and BC6), and one TNBC (BC5) (Table S1). We then subjected them to spatial ATAC-seq profiling, which was modified from microfluidic indexing-based spatial assay for transposase-accessible chromatin and RNA-sequencing (MISAR-seq) [22]. To achieve a profound investigation of the dynamic open chromatin, each spatial spot is 50 μm in diameter and roughly contained 10 cells. For spatial alignment of ATAC-seq signals and cell organization, each tissue section was stained with hematoxylin and eosin (H&E), and high-resolution anatomical images were captured. The distinct histological features were annotated by three professional pathologists blinded to the experiment. Spatial ATAC-based clustering and single-cell RNA sequencing (scRNA-seq) data integration were conducted to delineate the spatial expression profiles, regulatory element enrichment, and immune–tumor type interactions (**Figure 1**).

### Spatial ATAC-seq reveals regulatory commonalities across various BC patients and subtypes

**Figure 2:**
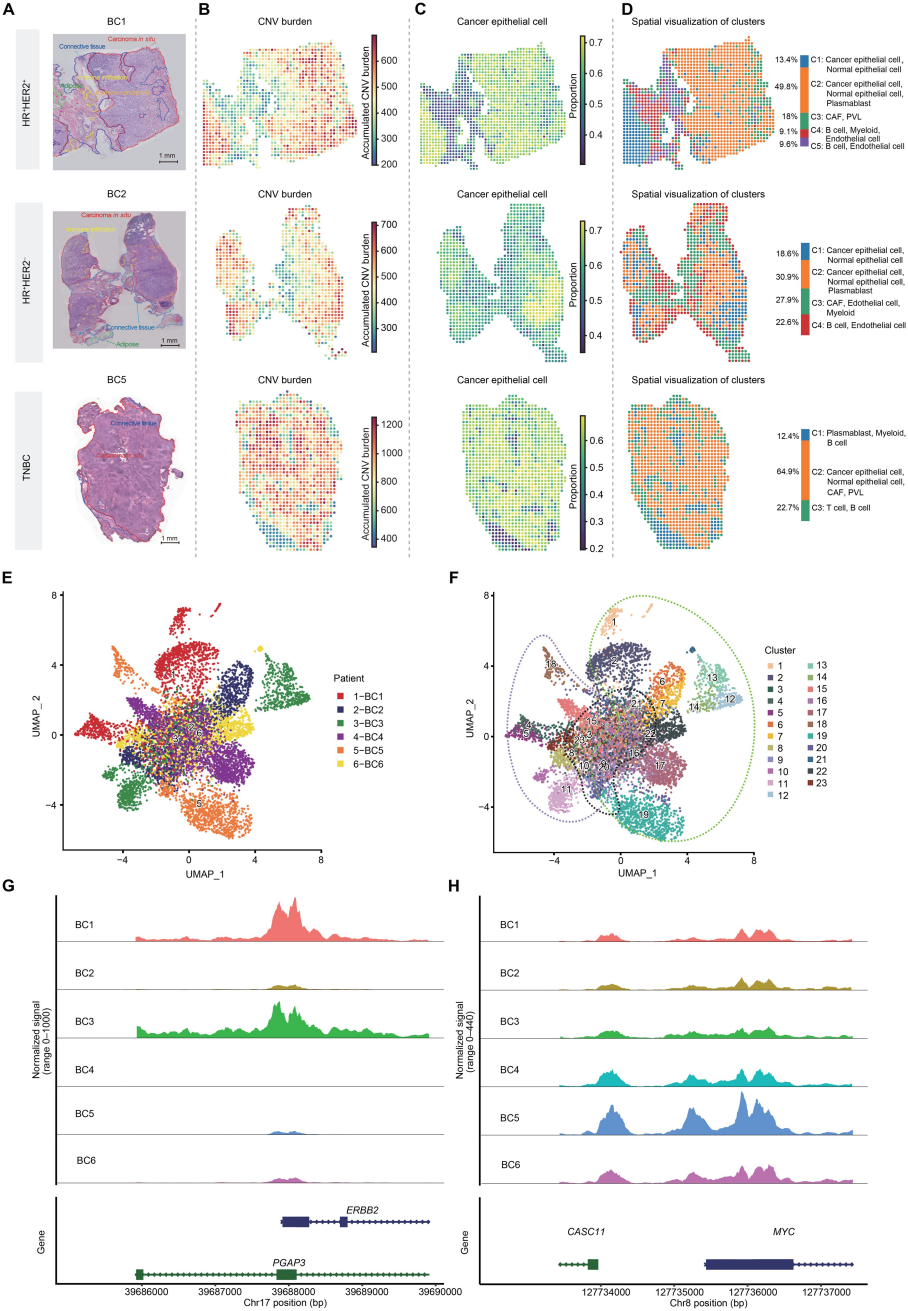
Spatial and genomic heterogeneity across BC samples **A**. Annotations made by three pathologists into five distinct categories (left): carcinoma *in situ* (red), connective tissue (blue), adipose (green), immune infiltration (yellow), and invasive carcinoma (orange). **B**. Spatial enrichment map of the accumulated CNV burden. **C**. Spatial visualization of cellular proportion of cancer epithelial cells. **D**. Spatial visualization of clusters for each patient. Spots with the same color belong to the same cluster. **E**. UMAP visualization of combined spots from BC samples, colored by patient ID. **F**. UMAP visualization of all integrated spots across all BC samples. Colors indicate integrated clusters. **G**. and **H**. Coverage plots showing normalized chromatin accessibility of key subtype-associated genes: *ERBB2* (G) and *MYC* (H). BC, breast cancer; UMAP, Uniform Manifold Approximation and Projection; CAF, cancer-associated fibroblast; PVL, perivascular-like cell.

We obtained high-quality accessible chromatin profiles (Figure S1). The number of spots per tissue section ranged from 1174 to 2237, and the median number of unique fragments per spot for each section varied between 19,599 and 33,937. We predicted the molecular subtype of each spot within the cancer region of all six patient samples using the previously published SCSubtype method [23]. Our study unveiled significant molecular heterogeneity in breast tumors beyond the classification by conventional immunohistochemistry (IHC) (Figure S2). At the current spatial resolution limit, where spatial spots contain several cells, the chromatin accessibility profile of a spot may represent a composite signal derived from a mixture of distinct cell types. To estimate the cell type composition of each spot, we employed transfer of annotations to cells, and their combinations (TACCO) [23] (with key modifications for compatibility with spatial ATAC-seq deconvolution, see Materials and methods), using a recently published large BC scRNA-seq dataset (GSE176078) [24] for cell type deconvolution. Specifically, we used the GeneScore, a computational estimate of a gene’s expression level based on chromatin accessibility of its regulatory elements by ArchR, as the input to integrate with the reference scRNA-seq dataset, based on the assumption that the chromatin accessibility of marker genes effectively represents the cell type signature within the mixture profile of each spot. The deconvolution analysis identified specific regions in each section with a high predicted density of tumor cells and characterized their spatial distribution of cell type-specific marker GeneScore (Figures S3 and S4). To validate our deconvolution method, we conducted a simulation analysis using bi-modal slide-tag libraries [25] from human melanoma samples, which included both single-nuclei ATAC-seq and single-nuclei RNA-seq (Figure S5A and B). The results demonstrated robust performance of TACCO deconvolution, which achieved accuracy comparable to that of RNA-based deconvolution, strongly supporting the validity of our method for spatial ATAC-seq deconvolution. In addition, to ensure the accuracy and reliability of the deconvolution strategy, we used an independent scRNA-seq reference dataset [26] for deconvolution. The consistent results across both references further validated our deconvolution methodology (Figure S5C). Additionally, there was a pronounced anti-correlation pattern between the composition of tumor cells and that of T cells, B cells, myeloid cells, and cancer-associated fibroblasts (CAFs), indicating a successful delineation of TME (**Figure 2**A–D, Figure S6). Of note, the spatial distribution of non-malignant cell types displayed significant heterogeneity both across and within individual sections (Figures S3 and S4). The correlation matrix depicted in Figure S7 demonstrates the high degrees of consistency between sample replicates.

Next, to further characterize the spatial distribution of the malignant tumor cells, we inferred the CNV for each section at 10-megabase (MB) genomic bin level (see Materials and methods). The cumulative CNV burden for each spot was quantified as the total number of CNV mutations within that location. As expected, regions identified as tumor areas in H&E staining exhibited a higher cumulative CNV burden than non-tumor regions (Figure 2B, Figure S6B). Furthermore, regions enriched with tumor epithelial cells consistently showed a significantly higher CNV burden across all samples (Figure S6E).

We then performed unsupervised clustering analysis using latent semantic indexing (LSI) embedding derived from the chromatin accessibility profile and identified three to five ATAC clusters for each section (Figure 2D, Figure S6D). All clusters for each section were meticulously annotated based on the H&E pathologists’ assessments (Figure 2A–D, Figure S6A–D), cellular composition, and cumulative CNV burden. The distribution of these clusters exhibited profound and distinctive spatial patterns. For example, spatially disjoint tumor clusters within the patients BC1 (Clusters 1 and 2) and BC2 (Clusters 1 and 2) showed distinct differences in their surrounding microenvironments. BC1 was characterized by noticeable heterogeneity, whereas BC2 was associated with a more uniform distribution of immune and stromal cell populations (Figure 2D). Additionally, Clusters 3 and 4 in BC3, which were spatially adjacent and enriched with epithelial cells, demonstrated varying levels of immune infiltration (Figure S6D). Next, we conducted differential analysis based on GeneScore [false discovery rate (FDR) ≤ 0.1 and log_2_ FC ≥ 0.5] to obtain a detailed view of the regulatory accessibility of breast cancer. Using the BC1 sample as an example, the differential regulatory element analysis enriched cluster specific gene activity modules (Figure S8), which recapitulated the characteristics of respective tissue regions. In general, clusters from different patients with similar identities exhibited high correlation (Figure S9). For example, epithelial cells from different patients shared the most similarity, whereas myeloid cells, endothelial cells, and CAFs were clustered together, and B cells, T cells, and plasmablasts showed higher correlation among themselves. Of note, no patient-specific clustering trees were found in the unsupervised clustering, supporting that the deconvolution was not confounded by patient-specific bias (Figure S9A).

To characterize the chromatin accessibility landscape of all BC samples, we performed integrative analysis on all sections of these six patients using Harmony and conducted unsupervised clustering, which showed 23 distinct clusters in Uniform Manifold Approximation and Projection (UMAP) (Figure 2E and F, Figure S9B). Notably, some clusters from all samples were characterized by higher immune and stromal cell type compositions and a lower cumulative CNV burden (Figure 2B and D). Additionally, the clear separation of these tumor clusters by various patients underscores the considerable epigenetic heterogeneity of cancer regions across different patients. Further investigation into promoter accessibility of PAM50 genes (a set of 50 specific genes used for classification of breast cancer into various molecular subtypes) revealed specific promoter accessibility for *ERBB2* [27] in HR^+^HER2^+^ tumors, while *MYC* [28], a key oncogene in breast cancer, exhibited striking variation in chromatin accessibility signals across patients, in line with their respective genetic features (Figure 2G and H). Substantial variations in accessibility signals among different samples are likely attributed to the presence of sample-specific CNVs [29].

Taken together, the spatial ATAC-seq landscape effectively captures the epigenetic heterogeneity across patients and tissue regions, allowing us to further explore the complex epigenomic landscape of human breast cancer.

### Spatial ATAC-seq reveals fundamental breast tumor regulatory features

**Figure 3:**
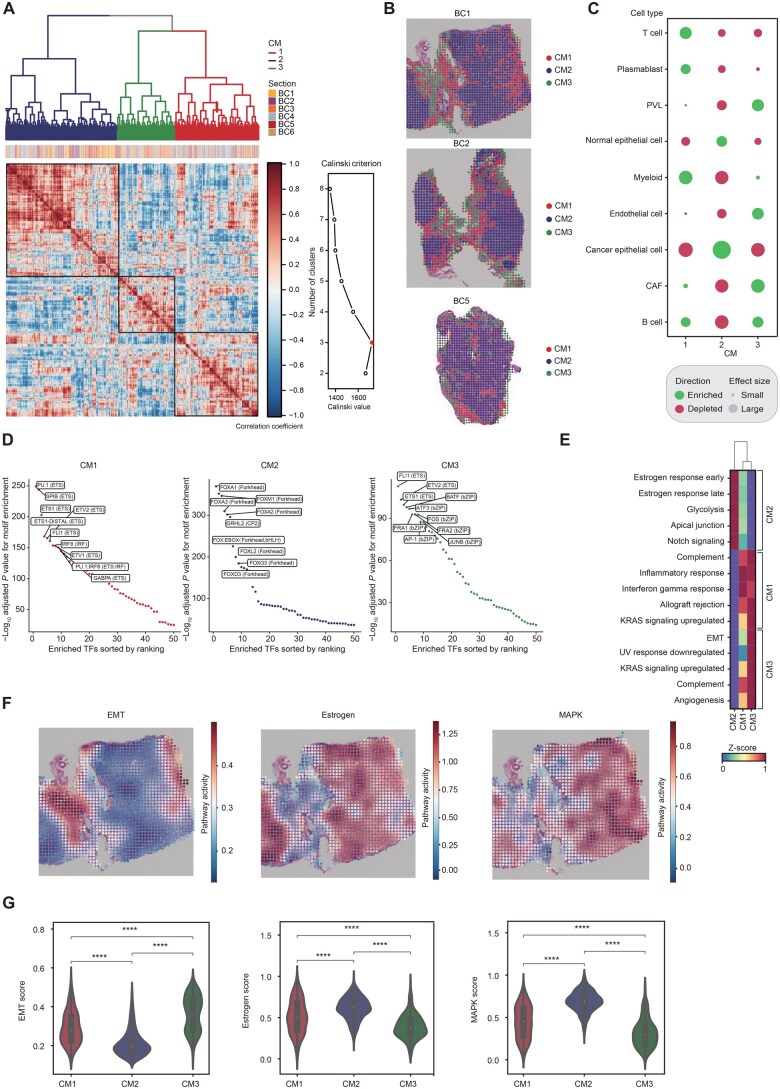
Comprehensive characterization of CMs in BC tissue samples **A**. Heatmap of the cell neighborhood clustering for all BC samples based on the cell type proportion in each spot. Hierarchical clustering (Ward.D2) and the Calinski criterion confirmed three modules with the best separation. **B**. Spatial visualization of CMs (CM1, CM2, and CM3) across three patients: BC1, BC2, and BC5. **C**. Cell type enrichment and depletion in CMs. Red indicates depletion, and green indicates enrichment. Marker size represents the effect size. **D**. Enriched motifs found by Homer in three cell modules: CM1, CM2, and CM3. **E**. GO term analysis for differentially accessible peaks of three CMs. Top five significant GO terms for each CM are listed. **F**. Spatial pattern of pathway scores: EMT, estrogen, and MAPK. **G**. Violin plots illustrating the distribution of EMT, estrogen, and MAPK pathway scores across CM1, CM2, and CM3 (*t*-test; ****, *P* < 0.0001). CM, cell module; GO, Gene Ontology; TF, transcription factor; EMT, epithelial–mesenchymal transition.

To explore the cellular heterogeneity of BC tissues, we performed the cell neighborhood clustering for all sections based on the cell type proportion in each spot (**Figure 3**A; see Materials and methods). Hierarchical analysis revealed three major cell modules shared in the six spatial ATAC-seq samples, known as Cell Module 1 (CM1), Cell Module 2 (CM2), and Cell Module 3 (CM3). These modules were located in distinct regions of BC sections, underscoring the profound cellular heterogeneity common to all cancer tissues (Figure 3B). These CMs can therefore be regarded as potential spatial niches with distinct functions for breast cancer.

To further elucidate differences in cell type distribution and intrinsic molecular programs across the modules, we performed enrichment analyses of cell type composition, TF motifs, and functional pathways for each module (Figure 3C–G). These analyses revealed that, in addition to providing a comprehensive depiction of spatial heterogeneity across tissue sections, CMs reflect detailed molecular programs that may account for common cellular mechanisms in breast cancer. For example, CM1 enriched in immune cells (T cells, B cells, and myeloid cells) showed infiltration within or adjacent to tumor regions (Figure 3C). Meanwhile, CM1 was characterized by its significant enrichment for TF motifs of the ETS family, further supporting its involvement in immune-related regulation (Figure 3D). The ETS family was reported to be aberrantly upregulated in solid tumors and associated with tumor growth, metastasis, and chemotherapeutic resistance via various mechanisms, including lineage specification and self-renewal of cancer progenitors, genome instability and DNA damage, epigenetics, and metabolism [30]. In contrast, CM3, which included perivascular-like cell (PVL), endothelial cell, CAF, and B cell, was marked by the enrichment of AP-1 family [31] TFs (*e*.*g*., BATF, ATF3, FOS, FRA2, and JUNB) (Figure 3D). The AP-1 family is well known for its importance in tumorigenesis and cell transformation to malignancy [32]. Its translational significance has been underscored by the potential of AP1 family members to serve as prognostic biomarkers for breast cancer [32] and therapeutic targets [33]. Functional enrichment analysis of CM3 specific chromatin features highlighted the tumor-related pathways, including the complement system, inflammatory response, interferon-gamma response, allograft rejection, KRAS signaling, and angiogenesis [34] (Figure 3E). These findings indicate that CM1 and CM3 represent distinct functional regulatory landscapes within the TME, with CM1 being immune-dominant and CM3 being stroma-driven.

On the other hand, CM2 exhibited significant enrichment in both normal epithelial and cancer epithelial cells, suggesting a predominant tumor identity for this module (Figure 3C). Motif enrichment analysis identified the notable presence of FOX family TFs [35], including FOXA1, FOXM1, FOXA2, FOXO3, FOXL2 [36], and FOXD3 [37] (Figure 3D). Importantly, the FOXA subfamily has been known to play a critical role in HR^+^HER2^+^ BC growth and progression [35,38]. Functional enrichment analysis of CM2 chromatin regions identified a range of tumorigenesis-related pathways, such as early and late-stage estrogen response, glycolysis, apical junction, and NOTCH signaling, highlighting the module’s critical role in tumor cell proliferation and interactions between epithelial and stromal cells (Figure 3E).

Given the highly consistent stratification of the tumor and TME across all patients, we further investigated whether there existed biological pathways or targets that can be generalized to breast cancer. We first investigated chromatin accessibility associated with the epithelial–mesenchymal transition (EMT) pathway, which plays a crucial role in tumor progression and metastasis [39]. Our results showed that tumor-related CM2 had the lowest EMT scores (Figure 3F and G), indicative of its high proliferative potential and epithelial characteristics, but low migratory capabilities. As expected, the pathway scores for MAPK [40] and Estrogen [41], hallmarks of BC progression, showed high enrichment in CM2 (Figure 3F and G). Upon further delineation of the spatial distribution of these pathways in BC1, we found that Estrogen and MAPK pathways showed notable variability within tumor-related CM2, in contrast to their presence in TME-related modules CM1 and CM3. These observations further indicate that a higher heterogeneity exists in tumor regions, which may account for resistance or low efficiency in cancer management.

To summarize, the CMs with distinct chromatin accessibility profiles identified across different patients by spatial ATAC-seq technology underscore the potential to distill the complex cancer phenotypes and genotypes into a provisional set of foundational principles, which may facilitate our understanding of the pan-mechanisms of BC development and malignant progression.

### Spatial ATAC-seq characterizes the tumor clonal heterogeneity

**Figure 4:**
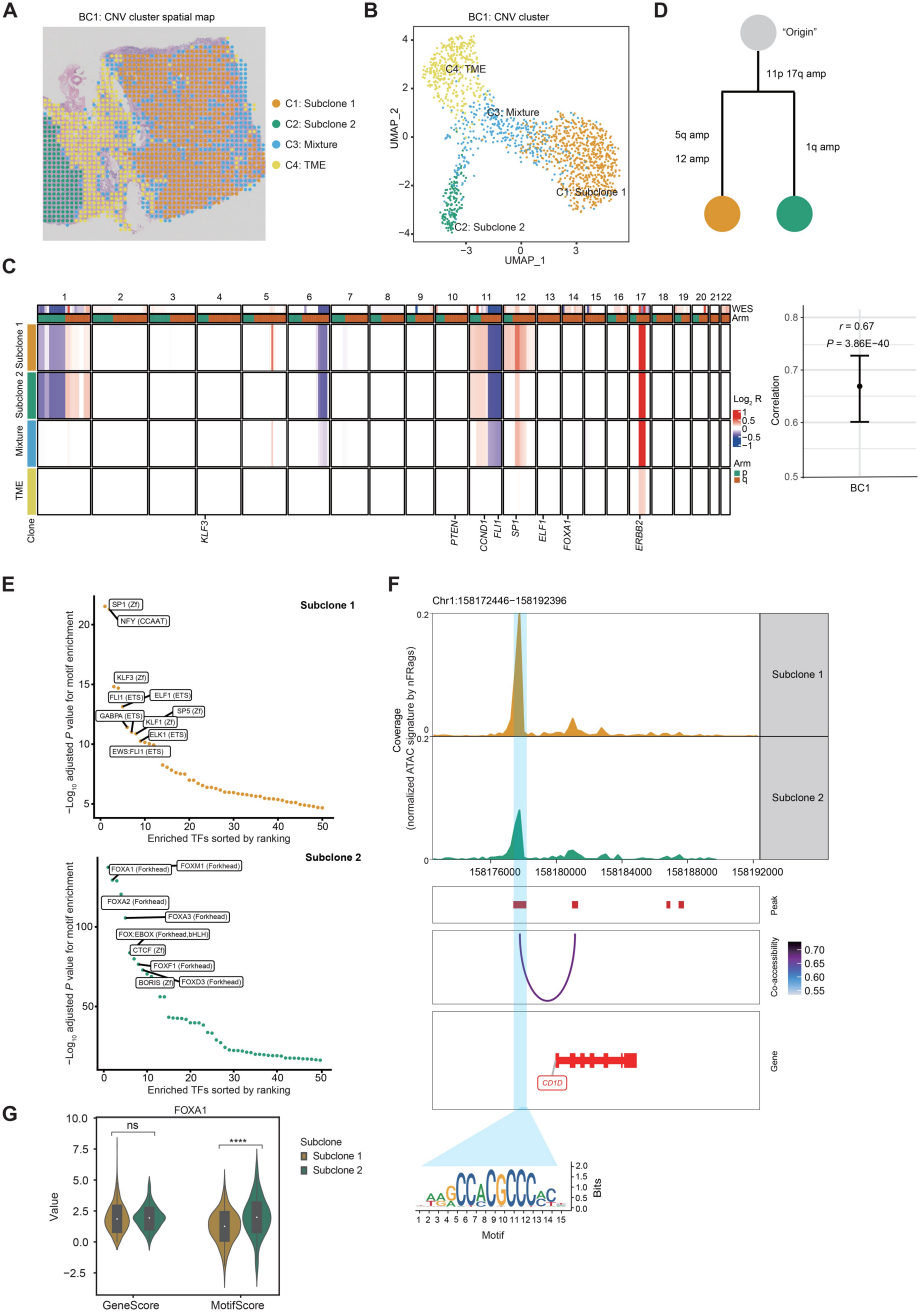
Spatial heterogeneity, TF enrichment, and chromatin regulatory dynamics in tumor subclones **A**. Spatial map of CNV clusters showing the distribution of C1 (subclone 1, orange), C2 (subclone 2, green), C3 (mixture, blue), and C4 (TME, yellow) across the tissue section. **B**. UMAP projection of CNV profile illustrating the separation of subclone 1 (C1), subclone 2 (C2), mixture (C3), and TME (C4). **C**. Left: Heatmap of the CNV signals (log_2_ R values) across subclones. Subclone 1 (C1) and subclone 2 (C2) exhibit distinct CNV patterns, while the mixture (C3) and TME (C4) show intermediate or stable profiles. Right: genome-wide CNV predictions from WES correlated with ATAC-seq estimates (*r* = 0.67, *P* < 0.05), indicating high concordance between the datasets. **D**. Evolutionary tree of the identified subclones from (C), inferred from CNV data. **E**. Motif enrichment analysis performed using Homer shows ETS family motifs (*e*.*g*., SP1) enriched in subclone 1, while Forkhead family motifs (*e*.*g*., FOXA1) are enriched in subclone 2. **F**. Chromatin accessibility and co-accessibility at the *CD1D* locus. Subclone 1 shows higher chromatin accessibility and co-accessibility at the *CD1D* locus (Chr1:158172446–158192396) than subclone 2. A zoomed-in region highlights an SP1 binding motif, suggesting its regulatory role in *CD1D* expression. **G**. Violin plots illustrating the distribution of GeneScore and MotifScore for FOXA1 across the two subclones. Subclone 2 shows a significantly higher MotifScore compared to subclone 1 (*t-*test; ****, *P* < 0.0001), while GeneScore shows no significant difference (ns, not significant). TME, tumor microenvironment; amp, amplification.

ITH contributes to the tumor progression and response to treatment. Here, we used BC1 as an exemplary case (**Figure 4**) to illustrate the clonal architecture and evolution in BC samples. We inferred genome-wide CNVs from spatially resolved ATAC-seq data, as CNVs deduced from ATAC-seq data are less affected by transcriptional regulation and preserve direct genomic structure. The analysis of spatial CNVs within tumor samples has unveiled crucial insights into the heterogeneity of cancer clonality. By systematically classifying CNV profiles into distinct clusters that recapitulate discernible histological features and CMs, we elucidated the intricate heterogeneity of tumor subclones. Four primary clusters were identified within the BC1 section, each characterized by unique CNV signatures that reflect the underlying biology of the tumor (Figure 4A and B). Clusters C1 and C2 were notably defined by CNVs exclusive to tumoral regions, suggesting the existence of spatially distinct tumor subclones, henceforth referred to as tumor subclones 1 and 2, respectively. In contrast, Cluster C3 showed CNV patterns indicative of mixed cellular components, whereas Cluster C4 was devoid of CNV features, aligning with the typical characteristics of the TME. To evaluate the robustness and accuracy of the CNV inference method, whole-exome sequencing (WES) was performed on the remaining patient samples (Figure 4C, Figures S11C and S12C). The results showed that the CNV profiles deduced from WES closely matched those from spatial ATAC-seq. Additionally, the CNV inference method was validated using spatial ATAC-seq data simulated from scATAC-seq datasets with known clonal identities [42] (Figure S10A–C). We also performed a visualization analysis of the relationships among clusters in Figure 2, CMs in Figure 3, and CNV subclusters in Figure 4 and Figure S10D. Three classifications captured distinct biological features and showed varying degrees of hierarchical overlap. Of note, both subclones 1 and 2 shared amplifications on chromosomes 11p and 17q and deletions on chromosomes 1p, 6q, and 11q, all of which are associated with increased malignancy risk [43–46] (Figure 4C). We inferred the clonal relationship of subclones C1 and C2, suggesting a common ancestral origin (Figure 4D). We identified unique CNV features for each subclone, such as amplification of 5q and 12 in subclone 1 and amplification of 1q in subclone 2. The observed differences between these subclones suggest phenotypic diversity, which may be associated with the heterogeneity in breast cancer. This heterogeneity could potentially contribute to treatment resistance in some BC cases. Heterogeneous CNV patterns were also observed in BC2–6 (Figures S11 and S12).

To further investigate the intrinsic differences in epigenetic regulatory elements between these subclones, we performed differential accessibility peak analysis. The featured peaks observed for each subclone exhibited distinct genomic distributions: subclone 1 demonstrated a higher enrichment of peaks localized to promoter regions, whereas subclone 2 displayed a broader distribution with a slight enrichment in distal regions (Figure S11D). Next, we compared motif enrichments on differential peaks between these two subclones using Homer (Figure 4E). We found an enrichment of SP1 [47,48] and NFY [49] motifs in subclone 1 and forkhead family motifs, such as FOXA1 and FOXM1 in subclone 2. Based on these findings, we investigated whether the enriched TF motifs could be linked to downstream effective targets. We observed higher activity levels of SP1 motifs near the *CD1D* gene promoter (a chromosome 1q gene), which also displayed higher accessibility in subclone 1 (Figure 4F, Figure S11E). Among subclone 2 enriched motifs, FOXA1 is one of the luminal-lineage TFs associated with BC proliferation, progression, and drug resistance [50]. We compared the GeneScore and MotifScore of FOXA1 between these two subclones (Figure 4G). Intriguingly, the GeneScore of *FOXA1* remained unchanged across tumor subclones, suggesting that particular gene expression metrics may be limited in discerning subtle differences among subclonal populations. In contrast, the MotifScore analysis unveiled elevated FOXA1 activity in subclone 2, indicative of the regulatory mechanisms that likely contribute to the adaptive properties of tumor cells. This contrast highlights the advantages of assessing spatial regulatory features over traditional gene expression methods to understand how subclonal heterogeneity influences tumor progression and response to therapy.

In addition to these regulatory activities, two subclones exhibited distinct signaling profiles. Subclone 1 showed higher MAPK activity (Figure S11F), which aligns with the observed enrichment of the SP1 motif, as SP1 plays a pivotal role in driving MAPK-related processes in breast tumors [51]. Meanwhile, subclone 1 showed higher mTOR signaling activity than subclone 2 (Figure S11G), suggesting potential crosstalk between the MAPK and the mTOR signaling pathways in driving tumor growth in subclone 1 (Figure S11H) [52]. In contrast, subclone 2 exhibited a pronounced estrogen signaling activity, as indicated by its significantly elevated estrogen score. The enhanced proliferation and invasive capacity of subclone 1, driven by MAPK signaling, contrasts sharply with the estrogen-driven characteristics of subclone 2. This difference not only elucidates the unique biological behaviors of each subclone but also emphasizes the necessity for tailored approaches in targeting these distinct signaling pathways in future research and clinical applications. Additionally, two distinct treatment strategies were employed for the BC1 case. Initially, hormone therapy was attempted but resulted in an inadequate tumor response and incomplete remission. Subsequently, DS8201 targeting HER2 therapy was administered. Spatial heterogeneity, reflected in differential enrichment of MAPK and estrogen pathways, likely underlies the variability in treatment responses. Furthermore, a survival analysis was conducted for a clinical database with varied expression levels of *SP1* and *CD1D* (Figure S13). The results indicated that patients with higher *SP1* expression were at risk. Similar trends were observed for *CD1D*, a potential downstream regulatory target of SP1. This association holds potential for the development of novel treatment strategies in the management of breast cancer. Spatial heterogeneity in tumors complicates cellular regulation and impacts treatment efficacy; therefore, treatment strategies should account for this heterogeneity to better target tumor complexity.

### Regulatory dynamics along continuous TME

In light of the varying degrees of CNV burden associated with these two distinct subclones, we sought to explore the TMEs of each subclone, which may convey specific phenotypes. The presence and activity of immune cells within the TME play a pivotal role in shaping the characteristics and BC progression [8,53]. The tumor border region, where malignant cells invade adjacent tissues and interact directly with various stromal cells and extracellular matrix (ECM) components, forms a complex and dynamic ecosystem [54–56]. Moreover, the tumor border area is critical for understanding processes such as tumor progression, immune surveillance, and disruption of normal tissue integrity.

**Figure 5:**
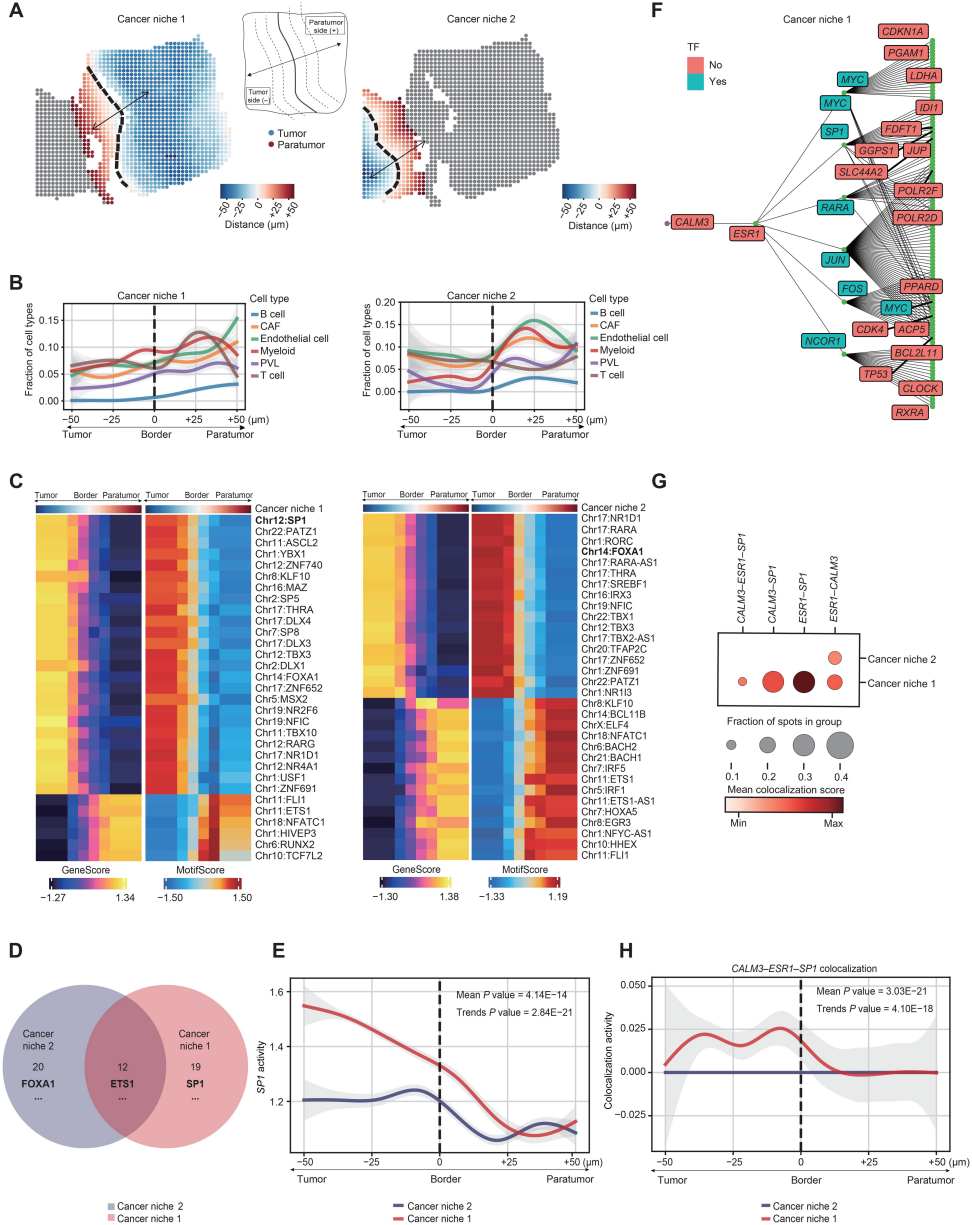
Spatial organization of the TME regulatory landscape along the cancer niche axis **A**. Construction of the tumor border in margin areas using spatial ATAC-seq data. Spatial distribution of tumor and paratumor regions in cancer niches 1 and 2, with color representing the distance from the tumor border. **B**. Line graphs showing the average fraction of different subsets along the tumor–border–paratumor axis of BC1 as determined using spatial ATAC-seq data. **C**. Heatmaps of TF regulators exhibiting a positive correlation between GeneScore and MotifScore in cancer niche 1 and cancer niche 2 across the tumor, border, and paratumor zones. Color intensity reflects GeneScore and MotifScore activity enrichment. **D**. Venn diagram showing the overlap of TF regulators between cancer niche 1 and cancer niche 2. The numbers represent the unique and shared TF regulators between cancer niches 1 and 2. **E**. *SP1* GeneScore across cancer niche 1 and cancer niche 2 along the spatial axis. Shaded areas represent confidence intervals, mean *P* value (*t*-test; *P* < 0.05), and trends *P* value (Granger causality test; *P* < 0.05), indicating statistical significance. **F**. Ligand–receptor–target signaling pathway analysis in cancer niche 1. Nodes in teal indicate TFs, while nodes in pink represent non-TF genes. Edges represent ligand–receptor–target interactions, with *CALM3* and *ESR1* at the center of the network. **G**. Dot plot showing the colocalization among *CALM3*, *ESR1*, and *SP1* across cancer niche 1 and cancer niche 2. Dot size represents the fraction of spots in each group, and the color intensity reflects the colocalization scores. **H**. Colocalization of *CALM3*, *ESR1*, and *SP1* across cancer niche 1 and cancer niche 2 along the spatial axis. Mean *P* value (*t-*test; *P* < 0.05) and trends *P* value (Granger causality test; *P* < 0.05) indicate significant differences.

To examine molecular features in these two spatially distinct tumor niches at the border region, we developed a model for the precise segmentation of margin areas around the invasive tumor fronts at defined directional distances along the tumor–stromal axis (**Figure 5**A). We denoted them as cancer niche 1 and cancer niche 2 based on their respective tumor contact with subclones 1 and 2. First, we explored the cellular compositions of these two niches. Our analysis revealed a significant enrichment of immune cells and fibroblasts on the paratumoral side of the border compared to the tumor side (Figure 5B), indicating a relatively immunosuppressive environment in tumor regions. As expected, pathways related to apoptosis, hypoxia, and glycolysis were activated on the tumor side of cancer niche 1 and cancer niche 2 (Figure S14A and B). Hypoxia decreased from the tumor core to the periphery, likely due to poor tumor vascularization, potentially causing apoptosis of TME cells, including lower B cells and myeloid cells levels in the tumor (Figure 5B). Tumor cells adapt by increasing glycolysis to sustain ATP production. Conversely, the activities of pathways related to the interferon-alpha response and interferon-gamma response were upregulated on the paratumoral side (Figure S14A and B). Notably, we observed a higher concentration of myeloid cells within cancer niche 1 at distances ranging from −50 μm to 0 μm, suggesting that cancer niche 1 was characterized by a greater degree of immune infiltration compared to cancer niche 2.

Next, we explored the potential upstream regulators of these heterogeneous regions by inferring the putative TFs enriched in the ATAC peaks. We found that TF regulators transition from the tumor to the paratumor in both cancer niche 1 and cancer niche 2 (Figure 5C). Comparative analysis of TF regulators between cancer niches demonstrated distinct molecular landscapes (Figure 5D). Approximately 39% (20 out of 51) of the TF regulators were specific to cancer niche 2, while approximately 37% (19 out of 51) were specific to cancer niche 1. Notably, the remaining 23% (12 out of 51) of TF regulators were shared between both cancer niches, indicating common TF driven TME changes toward tumor fronts, which may be promising general therapeutic targets for tumor intervention. Of particular interest, *SP1* enriched in cancer niche 1 showed a gradual down-regulation transiting from tumor to paratumor (Figure 5E). Mechanistically, we found that *SP1* [47,48] is a putative downstream target of *ESR1* in the cancer niche 1, suggesting that tumor cells may directly regulate tumor niches, therefore highlighting a sophisticated interplay between the tumor and immune cells in establishing the TME (Figure 5F). Further analysis revealed that *CALM3* is more strongly associated with myeloid and normal epithelial cells, whereas *SP1* showed the highest correlation with cancer epithelial cells (Figure S14C). These findings suggest that these molecules are intricately involved in the interactions between tumor and immune cells within the TME. Moreover, the molecular enrichment analysis confirmed the significant distribution of *CALM3*, *ESR1*, and *SP1* in cancer niche 1 cells, compared to that in cancer niche 2 (Figure 5G). Spatial colocalization mapping validated the specific enrichment of *CALM3*, *ESR1*, and *SP1* in cancer niche 1 along the tumor-paratumor axis (Figure 5H, Figure S14D).

Taken together, the spatial interactions between immune cells and tumor cells, particularly the regulatory cascade revealed through spatial ATAC-seq data, are instrumental in shaping the characteristics of different tumor niches and potentially influencing clinical outcomes. Spatial ATAC-seq data from BC can provide a powerful means for identifying regulatory elements that enable precise targeting of specific cell types.

## Discussion

The past decade has witnessed a paradigm shift in the field of biomedical research, primarily driven by the emergence of single-cell resolution transcriptomic technologies. These advances have allowed scientists to explore complex biological processes with unprecedented detail, particularly in cancer research. However, while transcriptomic analyses elucidate cellular behavior at a resolution unparalleled in earlier studies, the intricate regulatory epigenetic processes governing these behaviors remain to be thoroughly understood, especially when examined spatially. In this context, our study seeks to fill this critical gap by providing a comprehensive analysis of chromatin accessibility in human BC samples using a spatial omics approach.

Through the utilization of spatial ATAC-seq, we profiled the chromatin accessibility in BC tissues from six patients diagnosed with breast cancer. The spatial ATAC-seq platform was pivotal in revealing the spatial heterogeneity of the tumor environment, which is characterized by distinct modules including tumor, immune, and stromal cell types. The application of integrative bioinformatics techniques, such as clustering analysis, deconvolution, pathologist annotation, and functional enrichment/depletion analysis, has enabled us to delineate the ITH of breast cancer. Our results demonstrated a marked distinction among the aforementioned CMs, offering meaningful biological insights that can inform both prognostic and therapeutic strategies.

A significant aspect of our findings is the identification of CNV — a common and crucial form of structural variation observed in cancer genomes. CNV analysis has been widely studied in the context of scRNA-seq. However, similar methodologies for spatial ATAC-seq data remain notably scarce. Our study proposes a novel framework for CNV analysis derived from spatial ATAC-seq data, including a comprehensive multi-layered perspective integrating gene expression, chromatin feature distribution, and pathway enrichment, thereby broadening the toolkit available for researchers in cancer genomics. As tumors accumulate CNVs, they develop greater genomic diversity, leading to subclonal populations that contribute to tumor progression, aggressiveness, therapy resistance, and metastasis. Understanding these complex clonal architectures is crucial for developing targeted treatment strategies aimed at mitigating the impact of tumor diversity on patient outcomes. Our findings revealed clear spatial separation between the two tumor subclones with shared clonal CNVs indicating a potential common origin. Moreover, the distinct activity of regulatory elements, even under uniform transcriptional patterns, underscores the pivotal role of epigenetic regulation in driving tumor heterogeneity. Such advancements are essential, as the spatial context of chromatin accessibility profiles can influence tumor behavior and responsiveness to treatment.

Our study also revealed marked heterogeneity in regulatory activities between the two distinct cancer subclones. Each of these two separate subclones exhibited differential enrichment of TF motifs. Motifs of SP1 were highly enriched in subclone 1, whereas *FOXA1* was predominantly expressed in subclone 2. FOXA1 is primarily involved in estrogen-dependent signaling pathways. Upon binding to ERα, FOXA1 regulates the estrogen response element (ERE) on the genome, thereby promoting the expression of downstream genes such as *BCL2*, *MYC*, *PGR*, and *CCND1*. In contrast, SP1 is implicated in the MAPK signaling pathway. Binding of SP1 to ERK1/2 may facilitate crosstalk with the mTOR signaling pathway [52]. Interestingly, FOXA1 has been reported to play a dual role in BC depending on the hormonal status [38]. For example, FOXA1 expression leads to better prognosis in luminal cancers, but with an adverse outcome in TNBC. Consequently, in a neoadjuvant setting, FOXA1 may reflect a poor response in luminal BC, whereas it increases chemosensitivity in TNBC. Intriguingly, we have identified that BC1 did not show significant disease remission following hormone treatment, which coincided with FOXA1 enrichment in the partial BC1 biopsy. This implies that the patient BC1 had an incomplete response to the endocrine therapy and may require combinatorial treatment with mTOR inhibitors, such as everolimus.

The co-activation of hypoxia, glycolysis, and apoptosis pathways in cancer niche 1 and cancer niche 2 implies the presence of an immunosuppressive microenvironment. A declining trend in hypoxia signaling from the tumor core to the periphery suggests a hypoxic environment, likely resulting from aberrant tumor vascularization, which may induce apoptosis among cells in the TME, including a reduced fraction of B cells and myeloid cells in the tumor compared to the paratumoral regions (Figure 5B). Concurrently, tumor cells adapt to hypoxic conditions by enhancing glycolytic enzyme expression to maintain ATP production, as evidenced by the upregulation of glycolysis pathways. Meanwhile, the shift in the activity of IFN-α and IFN-γ pathways from the tumor core to the periphery suggests a transition from an immunosuppressive to an immune-activating state. Hypoxia in the tumor core may dampen IFN-α and IFN-γ levels [57,58], while the oxygen-rich paratumoral area showed heightened immune cell activity and IFN-α and IFN-γ expression. Our findings hint at a role for hypoxia in immune tolerance and underscore the need to further explore the hypoxia-cancer link. Further research is required to clarify these relationships within the TME.

During the exploration of molecular interactions within the TME, we uncovered a notable interaction between *ESR1* in tumor cells and *CALM3* in immune cells, as supported by our spatial colocalization analyses of *CALM3*, *ESR1*, and *SP1*. This finding underscores the potential pathways through which immune cells can remodel tumor heterogeneity, ultimately affecting the prognosis of patients with HR^+^HER2^+^ breast cancer. Such insights enhance our understanding of the tumor–immune landscape and point toward potential therapeutic targets that warrant further clinical exploration.

Nevertheless, our study has its limitations. The limited sample size constrains the depth of our analysis, despite the successful implementation and application of cross-sample integration algorithms. Our analysis focused mostly on the commonalities across different patients and subtypes. To bolster the credibility of our results, we incorporated publicly available datasets to augment our conclusions. With the increase in sample sizes, we could delve into the similarity and differential analysis across various subtypes and patients with statistical support. Besides, we introduced an approach that used scRNA-seq as a reference for deconvoluting spatially resolved ATAC-seq data. Although we used another independent scRNA-seq reference dataset [26] for deconvolution to ensure the accuracy and reliability of our deconvolution strategy, it still has limitations. This approach assumes that chromatin accessibility can effectively reflect cellular characteristics. However, in certain specific cell types or cell states, chromatin accessibility may not always align perfectly with gene expression patterns. To enhance accuracy, future analyses should consider spatial multi-modal datasets, such as MISAR-seq, which integrates both chromatin accessibility and gene expression data from the same individual cells. Furthermore, the absence of experimental validation poses a challenge to definitively establish causative relationships among the identified molecular interactions. Consequently, we advocate for further experimental investigations to validate our observations and confirm the mechanistic relevance of *CALM3* and *ESR1* in BC treatment.

Overall, our study represents a significant advancement in understanding the epigenetic landscape of BC through high-resolution spatial ATAC-seq. By unraveling the complexities of chromatin accessibility and its relationship with ITH, our study contribute to a more nuanced comprehension of BC biology. The insights gleaned from this research hold promise for developing targeted therapeutic strategies; however, they underscore the need for future studies to validate and expand on our findings. As we continue to unveil the molecular intricacies of cancer, it is imperative to integrate these approaches with clinical trials to bridge the gap between discovery and therapeutic application.

## Materials and methods

### Breast tumor samples

BC samples were obtained from the Guangdong Provincial People’s Hospital, Guangzhou, China. Histological evaluations of patient tumors were performed by pathologists for diagnostic purposes, including the assessment of tumor characteristics such as hormone receptor status, HER2 expression, and Ki67 expression. Detailed sample and pathological information are provided in Table S1. Tissue was isolated from each region for immediate embedding in OCT and for spatial ATAC-seq analysis.

### H&E staining

The adjacent frozen slide was warmed at room temperature for 10 min and fixed with 200 μl of 4% paraformaldehyde (Catalog No. 50-00-0, Sigma-Aldrich, St. Louis, MI) for 1 min. After decanting the paraformaldehyde, 200 μl of Harry’s Hematoxylin was added to the slide and incubated for 2 min. After washing with water, the tissue section was stained with 200 μl of differentiation solution (Catalog No. A3179, Sigma-Aldrich) for several seconds and rinsed with distilled water. The section was then incubated with 200 μl of blueing reagent (Catalog No. BL1007A, Biosharp, Beijing, China) for 1 min and rinsed with distilled water. Finally, the section was stained with 200 μl of eosin (Catalog No. G1108-500ml, Solarbio, Beijing, China) for 3 min and washed with distilled water. Stepwise dehydration by 70% ethanol for 1 min, 80% ethanol for 1 min, 95% ethanol for 1 min, and 100% ethanol for triple dehydration of anhydrous ethanol. After adding Xylene to the surface of the slide, the slides were sealed with neutral balsam (Catalog No. BL1519A, Biosharp). The stained tissue slide was examined immediately or stored at room temperature for future analysis.

### Spatial ATAC-seq library preparation

Freshly frozen or OCT-embedded tissues were cryosectioned at a thickness of 10 μm. Spatial ATAC-seq libraries were prepared according to the MISAR-seq protocol [22]. Briefly, we optimized the permeabilization condition and Tn5 tagmentation time. Libraries of patients BC1–BC3 were sequenced on a MGI-seq 2000 sequencer (BGI company, Shenzhen, China) using a paired-end mode (read1 75 bp, read2 75 bp, index5 8 bp, and index7 50 bp). Libraries of patients BC4–BC6 were sequenced on a Salus sequencer in paired-end mode (read1 75 bp, read2 75 bp, index5 8 bp, and index7 50 bp).

### Spatial ATAC data preprocessing

Raw FASTQ files were converted to Cell Ranger ATAC format (v2.1) for processing, and the human reference genome (GRCh38) was used for sequence alignment. The resulting fragments file was filtered to retain only spots within the tissue, which were manually selected based on H&E images.

The filtered fragment files were then converted into ArchRProject objects using ArchR (v1.0.2). For each tissue section, peak calling was conducted using MACS2 with the parameters (-g hs --nomodel --shift --100 --extsize 200). The peak sets from each section were merged using the reduce function to generate a unified peak set. Peak annotation and distribution visualization for each cluster were performed using the ChIPseeker package (v3.19).

Dimensionality reduction was performed using the addIterativeLSI function based on this union peak set. Individual clustering and UMAP visualization were conducted via the addCluster and addUMA functions with default parameters.

To account for batch effects, an integration analysis was performed using the method described in our previous paper [22], which combined the spatial relationship of neighbors and iterative feature selection to construct a unified embedding space for all tissue sections. To evaluate the integration accuracy and investigate relationships between each cluster across all tissue sections, hierarchical clustering was conducted at the individual cluster level in the integration embedding space.

We predicted the molecular subtype of each spot within the cancer region of all six patient samples by using a previously published method named SCSubtype [24]. Additionally, we used the CoveragePlot function from the Signac package (v1.14.0) to visualize the spatial ATAC-seq signal coverage in the selected regions.

### Spatial deconvolution

To deconvolute spatial ATAC-seq data, we utilized an optimal transport-based deconvolution method from the TACCO package (v0.3.0) with publicly available single-cell transcriptomic data as the reference. The cell-type labels were curated from the original publication. Some deconvolution methods originally developed for spatial RNA-seq deconvolution, such as cell2location and RCTD, also exhibit robust performance in spatial ATAC-seq, as demonstrated in this benchmark. Many of these methods typically rely on modeling the count data using a probabilistic distribution and require the raw counts matrix as input. In contrast, TACCO utilizes unbalanced optimal transport to transfer annotations without explicitly modeling the underlying count distribution, making it well-suited for our application. In our benchmarking analysis, deconvolution using scRNA-seq as the reference and GeneScore matrix as input outperformed the use of scATAC-seq references and achieved accuracy comparable to RNA-based deconvolution methods. The GeneScore matrix was computed using the default model in ArchR, followed by filtering to remove genes with no expression variability across cells and spots lacking non-zero gene values. The filtered matrix was used as the input for the deconvolution analysis. The deconvolution results were generated using the annotate function in the TACCO package, with the parameters “method = ‘OT’, assume_valid_counts = True” specified.

### Cellular niche clustering

To identify spatially distinct cellular niches, we applied hierarchical clustering based on the deconvoluted cell composition of each spot across all sections. The optimal number of clusters was determined using the Calinski-Harabasz [59] index.

The enrichment or depletion of cell types in relation to the cellular niche was analyzed as follows: First, we calculated the average proportion for each region, called the true average. Then, we shuffled the spot indices of the proportion estimates 10,000 times, while keeping the region labels the same. For each shuffle, we calculated the average proportion for each region, creating a set of shuffled averages. We then compared the true average with all the shuffled averages. Finally, we calculated the enrichment score by dividing the average difference by the standard deviation of the differences.

### Differential and enrichment analyses

We utilized the getMarkerFeatures function to account for differential chromatin accessibility peaks across clusters by adjusting the TSS enrichment score and fragment number bias. The resulting set of peaks was filtered using a threshold of “FDR ≤ 0.1 and Log_2_ FC ≥ 0.5” and subsequently imported into the rGREAT package (v2.5.4) for functional enrichment analysis. The Benjamini-Hochberg method was applied for multiple testing correction. Enriched terms were filtered with thresholds of HyperFdrQ < 0.05 and BinomFdrQ < 0.05. The top 15 Gene Ontology (GO) terms were selected for visualization purposes.

GeneScore differential analysis followed the same procedure but applied the GeneScore matrix instead. Pathway activity inference and biological term enrichment based on GeneScore were conducted using the decoupler-py package (v1.6.0). For pathway activity inference, the PROGENy database was employed, utilizing a multivariate linear model. Biological term enrichment was performed using the Molecular Signatures Database (MSigDB) with an over-representation analysis approach. The *t*-test was conducted to compare activity scores between different groups.

Motif enrichment analysis for differential accessibility peak sets was conducted using the “findMotifsGenome.pl” command from HOMER (v5), with the parameters “-size ‘given’ -len 8,10,12 -mask -nomotif” specified.

### CNV inference

To conduct CNV analysis, we adapted a methodological pipeline originally developed for DNA-seq data and further validated for our spatial ATAC-seq data [60]. Briefly, genomic fragment files were initially counted within 10-MB bins, followed by GC-content correction using modal regression, and bins with high GC content (GC > 0.8), low coverage (minimum read count > 1), or overlapping with the ENCODE hg38 blacklist were excluded. Following the filter, the resulting GC-corrected counts were then smoothed and segmented using a circular binary segmentation algorithm. The cumulative CNV burden for each spot was quantified by summing the smoothed counts. CNV clusters were identified using the default clustering method implemented in Seurat (v5.1.0), with the smoothed counts as input.

The WES data analysis followed the GATK Best Practices. FASTQ files were aligned to the GDC’s GRCh38 human reference genome using BWA-mem (v.0.7.17) with default settings. The resulting BAM files were sorted, and duplicates were marked using GATK MarkDuplicatesSpark. Somatic CNVs were identified using GATK (v4.1.9.0). Specifically, a panel of normals was then generated using all normal samples as input, and the GATK functions CollectReadCounts, followed by CreateReadCountPanelOfNormals. For tumor samples, reads that overlapped the target interval were counted using the GATK function CollectReadCounts. Tumor read counts were then standardized and denoised using the GATK function DenoiseReadCounts, with the panel of normals as input. Segments were modeled using ModelSegments, with denoised copy ratios as input. Finally, copy ratios for the segment regions were called using CallCopyRatioSegments.

### Building tumor–boundary–microenvironment axis

To establish a continuous spatial axis for capturing the spatial relationship between the tumor, its boundary, and the surrounding microenvironment, we first delineated the spatially disconnected tumor region. This involved identifying tumor areas distinct from adjacent tissue. We then calculated the radial distance of each spatial spot within the neighboring region relative to the tumor boundary, providing a quantitative measure of proximity for further analysis.

To reveal the regulatory drivers of these functional axes, we integrated GeneScore with motif accessibility to identify the positive TF regulators along each spatial axis. Spots were grouped into 10 sequential percentiles and smoothed with a window size of 5. The smoothed chromVAR TF deviations and GeneScore matrices were then correlated based on matched names, and TFs with a correlation greater than 0.75 were selected as positive regulators.

A generalized additive model (GAM) was applied to each feature to model the non-linear dynamic trends along the spatial axes. Furthermore, the *t*-test and Granger causality test were utilized to assess shifts in mean values and differences in trends between the axes.

### Spatial colocalization analysis

To calculate the colocalization score for two genes, we used weighted cosine similarity, a spatial-weighted version of cosine similarity, to assess the spatial relationships between two variables. For three-gene colocalization scores, we first computed the two-gene colocalization score for each pair. If all pairwise co-localization scores were greater than zero, we calculated the mean as the three-gene colocalization score; otherwise, the value was set to 0. The accuracy of this approach can be influenced by spatial data dropout, which may introduce false negatives in colocalization mapping. Such a dropout diminishes detectable signals, especially in regions of low expression or sparse coverage, hindering the identification of true spatial relationships. Furthermore, heterogeneous cell density and tissue complexity can obscure or mimic spatial patterns, further complicating accurate colocalization analysis.

## Supplementary Material

qzag001_Supplementary_Data

## Data Availability

The raw sequence data reported in this study have been deposited in the Genome Sequence Archive for Human [61] at the NGDC, CNCB (GSA-Human: HRA008910), which are publicly accessible at https://ngdc.cncb.ac.cn/gsa-human.

## Code availability

The code has been submitted to BioCode at the National Genomics Data Center (NGDC), China National Center for Bioinformation (CNCB) (BioCode: BT007984), which is publicly accessible at https://ngdc.cncb.ac.cn/biocode/tool/BT007984.

## CRediT author statement

**Yingying Qian:** Investigation, Visualization, Validation, Writing – original draft, Writing – review & editing. **Miao Zhu:** Data curation, Formal analysis, Visualization, Writing – original draft. **Chongyang Ren:** Resources, Validation. **Yeyong Zhou:** Resources. **Jian Xu:** Resources. **Guangyu Zhang:** Writing – review & editing. **Liang Dong:** Writing – review & editing. **Cheukfai Li:** Resources. **Jiaoyi Lv:** Resources. **Qiaorui Xing:** Supervision, Writing – review & editing. **Guochun Zhang:** Conceptualization, Resources. **Guangdun Peng:** Conceptualization, Resources, Supervision, Project Administration, Writing – review & editing, Funding acquisition. **Ning Liao:** Resources, Supervision, Writing – review & editing, Funding, Acquisition. All authors have read and approved the final manuscript.
