# Supplementary material for: Spatial Chromatin Accessibility Analysis of Intratumor Heterogeneity in Breast Cancer
Source: Genomics Proteomics Bioinformatics. 2026 Jan 12;24(1):qzag001. doi: 10.1093/gpbjnl/qzag001 (PMC13457082; doi:10.1093/gpbjnl/qzag001)
Supplement: Supplementary file 1 [file qzag001_supplementary_data.zip › Supplementary material captions.docx]

**Supplementary materials**

**Figure S1 Quality assessment of spatial ATAC-seq data**

**A.** TSS enrichment profiles. **B.** Size distribution nsert fragments. **C.** TSS enrichment score versus Log_10_ (number of unique fragments) per spot in six BC patients.

**Figure S2 Distribution of BC subtypes across patients**

The bar graph shows the proportional composition of four breast cancer subtypes, Basal (blue), HER2E (orange), LumA (green), and LumB (red), within each sample. HER2E, HER2-enriched; LumA, Luminal A; LumB, Luminal B.

**Figure S3 Spatial mapping of cell types**

Spatial mapping of cellular proportion of various cell types in BC1 to BC6 samples, colored by cellular proportion of each cell type. PVL, perivascular-like cell; CAF, cancer-associated fibroblast.

**Figure S4 Spatial mapping of marker genes**

Spatial mapping of marker genes across BC1 to BC6 samples: *EPCAM* (epithelial cell), *MKI67* (proliferating cell), *CD3D* (T cell), *CD68* (myeloid), *MS4A1* (myeloid), *JCHAIN* (plasmablast), *PECAM1* (endothelial cell), and *PDGFRB* (mesenchymal cells: fibroblast/perivascular-like cells). Color represents GeneScore of the respective gene.

**Figure S5 Validation of deconvolution strategy**

**A.** Schematic of deconvolution strategies for spatial omics datasets. **B.** Left: Simulation of spatial multi-modal data derived from Slide-tags of a metastatic human melanoma sample [25]. Middle: JSD metric used to assess deconvolution accuracy. Right: RMSE metric used to compare deconvolution performance. T_reg_ cell, regulatory T cell; mDC, myeloid dendritic cells; Mono-mac, monocyte-derived macrophages; pDC, plasmacytoid dendritic cells; JSD, Jensen-Shannon Divergence. **C.** Left: UMAP visualization of two published scRNA-seq reference datasets for spatial deconvolution [24] [26]. Right: Correlation heatmap of the resulting cell composition.

**Figure S6 Spatial and genomic heterogeneity across BC samples**

**A.** Annotations made by three pathologists into five distinct categories: carcinoma in *situ* (red), connective tissue (blue), adipose (green), immune infiltration (yellow), and invasive carcinoma (orange). Scale bar, 1000 μm. **B.** Spatial visualization of accumulated CNV burden. **C.** Spatial visualization of cellular proportion of cancer epithelial cell. **D.** Spatial visualization of clusters for each patient. Spots with the same color belong to the same cluster. **E.** Box plots showing CNV burden in epithelial-enriched versus other regions across six BC samples (BC1–BC6). Green represents epithelial-enriched and orange represents others. *P* value indicate significant differences (*P* < 0.0001).

**Figure S7 Reproducibility analysis for BC5 and BC6 samples**

**A.** Left: Spatial visualization of clusters for patient BC5 replicates. Right: Correlation matrix showing the similarity in CNV profiles estimated from spatial ATAC-seq data of BC5 and its replicates, as well as that predicted from WES data. Correlation coefficients range from 0.90 to 1.00, indicating high concordance among samples. **B.** Left: Spatial visualization of clusters for patient BC6 replicate. Right: Correlation matrix showing the similarity in CNV profiles estimated from spatial ATAC-seq data of BC6 and its replicate, as well as that predicted from WES data. Correlation coefficients range from 0.88 to 0.99, indicating high concordance among samples. WES, whole-exome sequencing; rep, replicate.

**Figure S8 Characterization of the genomic and functional heterogeneity observed in BC1 clusters**

**A.** Heatmap of marker genes across patient BC1 clusters (C1–C5). Z-score indicate relative GeneScore levels. **B.** Heatmap showing differentially accessible peaks across patient BC1 clusters (C1–C5). Z-score indicate accessible levels. **C.** Heatmap showing the functional pathways highly enriched across patient BC1 clusters. Top five significant GO terms for each cluster are listed. EMT, epithelial–mesenchymal transition; GO, gene ontology. **D.** Bar plot showing the percentage distribution of chromatin features across patient BC1 clusters (C1–C5). Features include promoter (≤ 3 kb), 3' UTR, 5' UTR, 1^st^ exon, other exon, 1^st^ intron, other intron and distal intergenic region. **E.** Dot plot showing GREAT enrichment analysis for differentially accessible peaks in patient BC1 cluster C2. Dot size indicates the proportion of observed genes to total genes (Observed genes / Total genes), and color represents adjusted *P* values [Binomial fold enrichment (Observed / Expected)].

**Figure S9 Integration analysis of all patient samples**

**A.** Heatmap colored by Pearson correlation coefficients derived from the mean cellular proportions of each cell type across clusters. Three superclusters are highlighted, each characterized by similarities in their cellular proportions. Super cluster 1 (green): immune; Super cluster 2 (pink): connective tissue; Super cluster 3 (purple): stroma tissue. **B.** UMAP visualization of the integrated spots from all patient samples, colored by super clusters.

**Figure S10 Validation of CNV analysis**

**A.** Schematic of spatial ATAC-seq dataset simulation generated from a scATAC-seq dataset with validated clone identities [42]. **B.** Left: UMAP visualization of CNV profile-based clustering in the simulated spatial ATAC-seq dataset [42]. Right: Heatmap of CNV signals across clusters [42]. **C.** Left: Spatial visualization of ground truth clone identities in the simulated dataset [42]. Right: Spatial visualization of unsupervised clusters derived from inferred CNV profiles [42]. **D.** Sankey plot illustrating the relationships among unsupervised clusters, CMs, and CNV subclone identities in BC1. **E.** Cell type enrichment and depletion within CNV subclones. Red is indicative of depletion and green is of enrichment. Marker size represents the effect size. DC, dendritic cells; Amp, amplification; Del, deletion; LOH, Loss of Heterozygosity; CMs, cell modules; TME, tumor microenvironment.

**Figure S11 Spatial and genomic characterization of CNV clusters in BC3 and BC1**

**A.** Spatial map of BC3 CNV clusters showing the distribution of C1 (subclone 1, green), C2 (subclone 2, blue), and C0 (TME, yellow) across the tissue section. **B.** UMAP projection of CNV profile of BC3 CNV clusters, illustrating the separation of subclone 1 (C1), subclone 2 (C2), and TME (C0). **C****.** Left: Heatmap of CNV signals (log_2_ R values) across BC3 CNV clusters. Subclone 1 (C1) and subclone 2 (C2) exhibit distinct CNV patterns, while the TME (C0) shows stable profiles. Right: Genome-wide CNV predictions from WES correlated with ATAC-seq estimates (*r* = 0.89; *P* < 0.05), indicating high concordance between the datasets. **D.** Bar plot showing the percentage distribution of chromatin accessibility features in BC1 subclone 1 and subclone 2. **E.** Chromatin accessibility at the *SP1* locus in BC1 subclone 1 and subclone 2. ATAC-seq signal coverage at the *SP1* locus (Chr12:53355175–53405176) indicates higher accessibility in subclone 1 than subclone 2. **F.** Estrogen and MAPK pathway activity scores in BC1 subclone 1 and subclone 2. Violin plots show significantly higher estrogen scores in subclone 2 and higher MAPK scores in subclone 1 (*t* test; ****, *P* < 0.0001). **G．**Top: Spatial pattern of MTORC pathway activity scores in BC1 section. Bottom: Violin plots show significantly higher MTORC scores in BC1 subclone 1 (*t* test; **** *P* < 0.0001). **H.** Hypothesized MAPK–mTOR pathway crosstalk model.

**Figure S12 Spatial and genomic characterization of CNV clusters in BC2 and BC4**–**6**

**A.** Spatial map of CNV clusters in BC6, showing the distribution of C0 (subclone 1, orange), C1 (TME, blue), and C2 (subclone 2, green) across the tissue section. **B.** UMAP projection of CNV profile in BC6, illustrating the separation of C0 (subclone 1, orange), C1 (TME, blue), and C2 (subclone 2, green) across the tissue section. **C.** Left: Heatmap of CNV signals (log_2_ R values) across BC6 CNV clusters. Subclone 1 (C0) and subclone 2 (C2) exhibit distinct CNV patterns, while the TME (C1) shows intermediate or stable profiles. Right: Genome-wide CNV predictions from WES correlated with ATAC-seq estimates (*r* = 0.88; *P* < 0.05), indicating high concordance between the datasets. **D.**–**F.** Left: Spatial map of CNV clusters showing the distribution of C0 (TME, orange) and C1 (Clone, blue) across the tissue sections. Middle: UMAP projection of CNV profile in BC2 (D), BC4 (E), and BC5 (F). Right: Heatmap of CNV signal (log_2_ R values) across clones in BC2 (D), BC4 (E), and BC5 (F).

**Figure S13 Survival analysis of *SP1* & *CD1D* in BC patients**

**A.** Kaplan-Meier curve illustrating survival probability based on *SP1* expression levels in BC patients. High expression of *SP1* is associated with low survival outcomes (HR = 0.51, 95% CI: 0.28–0.91, Log_rank *P* = 0.021). **B.** Kaplan-Meier curve illustrating survival probability based on *CD1D* expression levels in BC patients. High expression of *CD1D* is associated with low survival outcomes (HR = 0.25, 95% CI: 0.13–0.48, Log_rank *P* = 7.1e-6).

**Figure S14 Characterization of cancer niches and validation of spatial colocalization**

**A.** Line graphs showing pathway enrichment scores for key cancer hallmarks in cancer niche 1, including angiogenesis, apoptosis, glycolysis, G2M checkpoint, EMT, inflammatory response, inflammatory gamma response, interferon alpha response, and hypoxia. **B.** Line graphs showing pathway enrichment scores for key cancer hallmarks in cancer niche 2, similar to A. **C.** Global bivariate correlation scores of *CALM3* (left) and *SP1* (right) across different cell types. **D.** Spatial colocalization scores for *CALM3*, *ESR1*, and *SP1* across tissue sections. The color scale represents the colocalization score, ranging from −1.0 to 1.0.

**Table S1 Patient information**
