# Supplementary material for: Spatial Chromatin Accessibility Analysis of Intratumor Heterogeneity in Breast Cancer
Source: Genomics Proteomics Bioinformatics. 2026 Jan 12;24(1):qzag001. doi: 10.1093/gpbjnl/qzag001 (PMC13457082; doi:10.1093/gpbjnl/qzag001)

Supplementary Figure 13

A

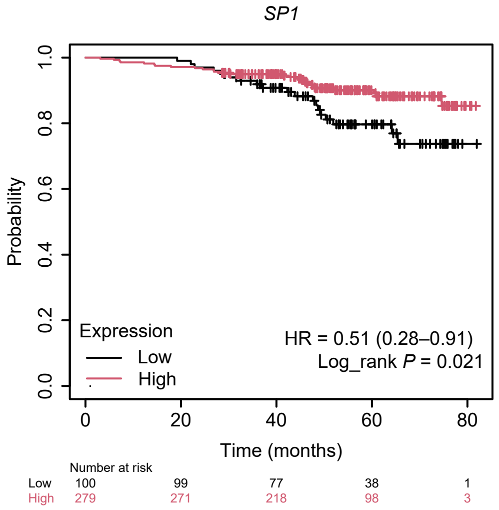

B

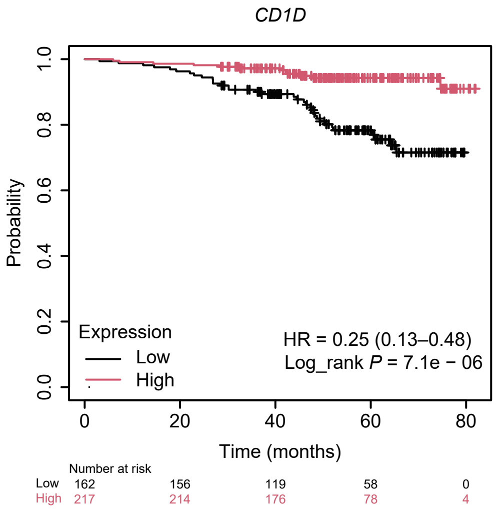

Supplement: Supplementary file 1 [file qzag001_supplementary_data.zip › Figure S13.pdf]
