# Supplementary material for: Spatial Chromatin Accessibility Analysis of Intratumor Heterogeneity in Breast Cancer
Source: Genomics Proteomics Bioinformatics. 2026 Jan 12;24(1):qzag001. doi: 10.1093/gpbjnl/qzag001 (PMC13457082; doi:10.1093/gpbjnl/qzag001)
Supplement: Supplementary file 1 [file qzag001_supplementary_data.zip › Table S1.docx]

| **Sample ID** | **Subgroup** | **Gender** | **Age at initial**  **pathological diagnosis** | **ER%** | **PR%** | **Ki67%** | **ER**  **status** | **PR**  **status** | **Hormone**  **receptor**  **status** | **HER2 immunohistochemistry level** | **HER2 final status** | **Experiment** |
| --- | --- | --- | --- | --- | --- | --- | --- | --- | --- | --- | --- | --- |
| BC1 | HR^+^HER2^+^ | Female | 46 | 90 | 90 | 20 | Positive | Positive | Positive | 3+ | Positive | Spatial ATAC-seq |
| BC2 | HR^+^HER2^–^ | Female | 60 | 60 | 5 | 20 | Positive | Positive | Positive | 1+ | Negative | Spatial ATAC-seq |
| BC3 | HR^+^HER2^+^ | Female | 48 | 95 | 100 | 30 | Positive | Positive | Positive | 3+ | Positive | Spatial ATAC-seq |
| BC4 | HR^+^HER2^–^ | Female | 59 | 100 | 60 | 30 | Positive | Positive | Positive | 2+ | Negative | Spatial ATAC-seq |
| BC5 | HR^–^HER2^–^ | Female | 61 | 0 | 0 | 80 | Negative | Negative | Negative | 0 | Negative | Spatial ATAC-seq |
| BC6 | HR^+^HER2^–^ | Female | 38 | 90 | 50 | 10 | Positive | Positive | Positive | 2+ | Negative | Spatial ATAC-seq |

**Table S1 Patient information**
